# A double-Flp-in method for stable overexpression of two genes

**DOI:** 10.1038/s41598-020-71051-5

**Published:** 2020-08-20

**Authors:** Ole Jensen, Salim Ansari, Lukas Gebauer, Simon F. Müller, Kira A. A. T. Lowjaga, Joachim Geyer, Mladen V. Tzvetkov, Jürgen Brockmöller

**Affiliations:** 1 Institute of Clinical Pharmacology, University Medical Center Göttingen, Georg-August University, Robert-Koch-Str. 40, 37075 Göttingen, Germany; 2 Institute of Pharmacology and Toxicology, Faculty of Veterinary Medicine, Justus Liebig University Giessen, 35392 Giessen, Germany; 3 Institute of Pharmacology, Center of Drug Absorption and Transport (C_DAT), University Medical Center Greifswald, 17489 Greifswald, Germany

**Keywords:** Genetic vectors, Transfection, Gene expression, Pharmacokinetics, Genetic vectors, Transfection

## Abstract

Overexpression of single genes in mammalian cells is widely used to investigate protein function in basic and applied biosciences and in drug research. A better understanding of interactions of two proteins is an important next step in the advancement of our understanding of complex biological systems. However, simultaneous and robust overexpression of two or more genes is challenging. The Flp-In system integrates a vector into cell lines at a specific genomic locus, but has not been used for integration of more than one gene. Here we present a modification of the Flp-In system that enables the simultaneous targeted integration of two genes. We describe the modification and generation of the vectors required and give the complete protocol for transfection and validation of correct genomic integration and expression. We also provide results on the stability and reproducibility, and we functionally validated this approach with a pharmacologically relevant combination of a membrane transporter facilitating drug uptake and an enzyme mediating drug metabolism.

## Introduction

The use of immortal cell lines has become an indispensable tool in basic and applied biomedical research in the last decades. In vitro experiments with cell lines are often used to generate new hypotheses, or to validate in vivo findings with the possibility to manipulate under well-defined conditions^1,2^. In drug research, cell lines overexpressing specific genes are an important screening tool. Overexpression can be achieved via numerous ways, with transient or stable expression of the gene of interest^3,4^. However, many transgene-introducing techniques come along with obvious disadvantages: Reproducibility, efficiency, and anisogeneity are only a few of them. Stable transfection, based on non-viral site-specific recombination circumvents these disadvantages^5–8^. This can be accomplished by the use of recombinases, such as Cre from the P1 bacteriophage or Flp from *Saccharomyces cerevisiae*. These enzymes can catalyze the recombination of two DNA strands at specific recognition sequences, making it possible to insert, excise, invert, or translocate DNA segments^9,10^. The Flp-In system (Thermo Fisher Scientific, Darmstadt, Germany) uses the Flp recombinase to generate isogenic cell lines, in which the gene of interest integrates at a single well-defined locus within the host cell genome^11–15^. One highlight of the Flp-In system is the fact that the promoter, which drives the expression of the resistance against the selection antibiotic hygromycin, is present upstream of the recombination site in the host cell genome. This ensures the expression of the resistance gene only in successfully transfected cells that carry and stably overexpress the gene of interest. Since the promoter and the locus of genomic integration are the same for all constructed cell lines, the expression level of the genes of interest and general isogeneity are well comparable^11^.

**Figure 1:**
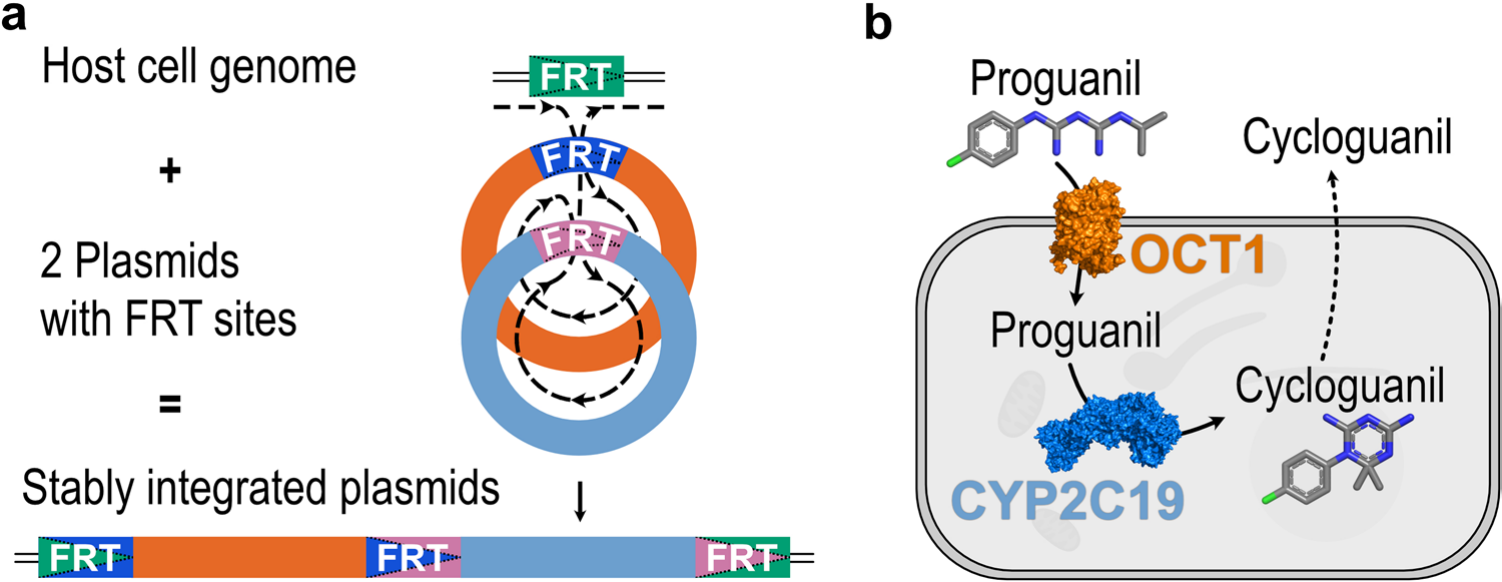
The principle and an application example of the Double-Flp-In technique. (**a**) The integration of two simultaneously transfected plasmids encoding two genes of interest can be achieved into a single Flp recombination target (FRT) site. (**b**) As a proof of concept, we transfected HEK293 cells with the coding sequences for the human organic cation transporter 1 (OCT1) and cytochrome P450 isoform 2C19 (CYP2C19). Together, these proteins will facilitate the uptake of the anti-malarial prodrug proguanil and the metabolism to its active metabolite cycloguanil. Protein icons of OCT1 and CYP2C19 and chemical structures were created using The PyMOL Molecular Graphics System, Version 1.3, Schrödinger, LLC, www.pymol.org.

The Flp-In system, so far, was not applicable when two or more genes of interest are to be overexpressed in a controlled manner, although that option actually exists. The Flp recombination target (FRT) site not only persists after the integration of a vector, but even a second FRT site is introduced upon vector integration (Fig. 1a). Modifying the Flp-In vector with internal ribosomal entry sites (IRES) would be one option to integrate and overexpress several genes^16^, but the expression efficiency has been shown to depend strongly on the distance between IRES element and translation start site. The expression of the genes is highly dependent on the order in the operon and requires a precise design^17,18^. The simple transfection of two kinds of the same vector is also a feasible approach^19^. However, without a second selection antibiotic resistance gene, there is no easy way to select cell clones successfully transfected with both vectors. Excessive and sophisticated validation would be needed to ensure the presence of both genes of interest and their expression levels.

**Figure 2:**
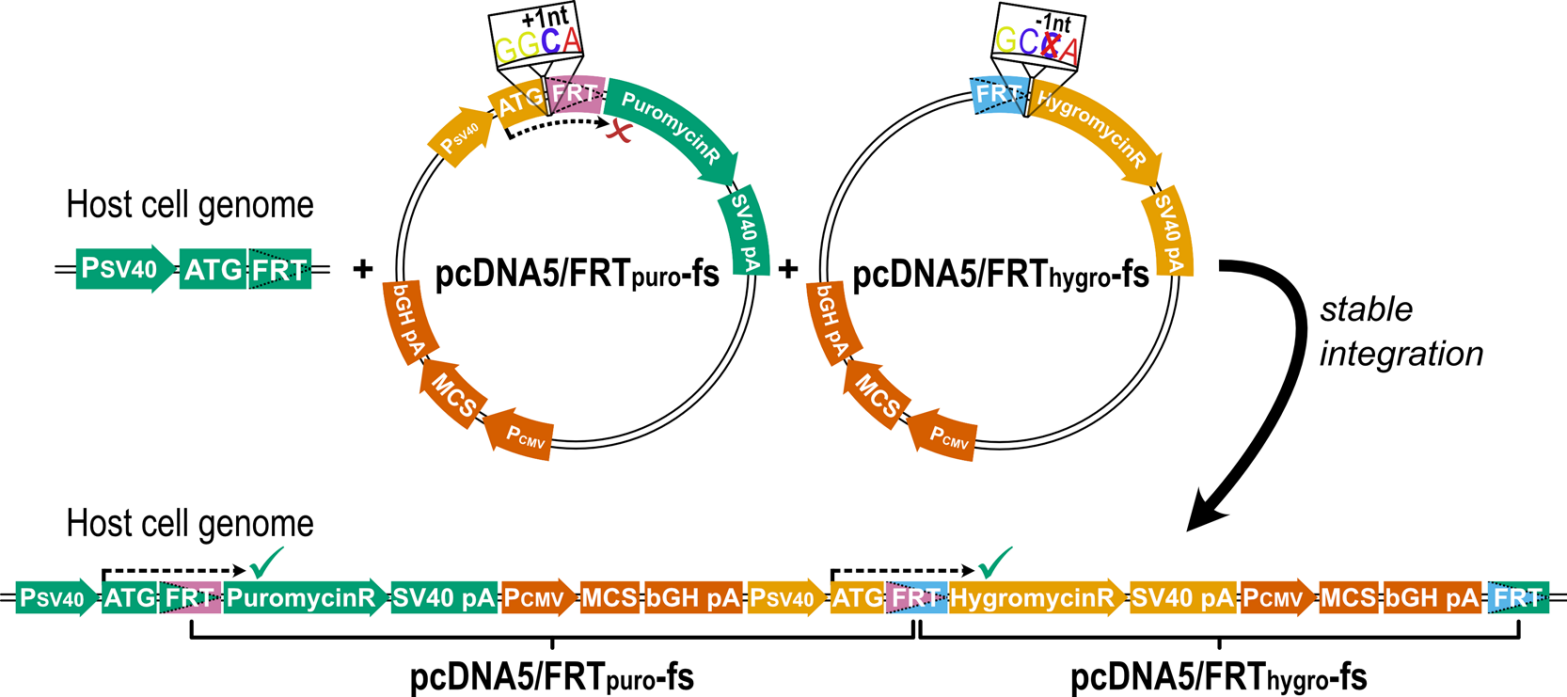
Illustration of the total construct and the reading frame shift introduced to ensure expression of both resistance genes only in case of successful double transfection. The reading frame shift introduced into the both FRT vectors leads to expression of both resistance genes only after successful stable integration (green ticks), and not before (red x). The introduction of an additional C between the start codon (ATG) and the FRT site is indicated. This allows reliable selection of double-transfected cell clones (sequence elements for bacterial expression are not shown) and expression of genes of interest cloned into the multiple cloning sites (MCS) from identical promoters. (*PSV40* SV40 promoter, *SV40 pA* polyA signal of SV40, *PCMV* CMV promoter; *bGH pA* polyA signal of bovine growth hormone).

Our goal was to develop a simple and robust protocol to transfect two different genes in a modular concept (Fig. 1). To achieve this, we introduced a one-nucleotide deletion into the original pcDNA5/FRT vector, which was necessary for correct expression of the hygromycin resistance gene from the promoter of the second vector. The second vector did not only include the promoter to drive hygromycin expression after correct integration but also the gene for puromycin resistance. To exclude that the puromycin resistance was already expressed upon transient presence of the vector, we inserted one nucleotide between the translation start site and the coding sequence of the puromycin resistance gene, which leads to a frame shift and to early termination of translation. After successful integration in the correct order, the deletion and the insertion will neutralize themselves and expression of both resistance genes will ensure proper selection (Fig. 2). These vectors can be used for the easy and replicable transfection of two genes for multifarious applications to study interactive actions of almost any two proteins. As proof of concept, we show the stability of the double transfection with two pairs of fluorescent proteins, we compare expression levels of single- to double-transfected genes, and we provide a scientifically relevant example for the application of this method to study the interaction between cell uptake transport and metabolism of the anti-malarial prodrug proguanil (Fig. 1). The scope of this technique, however, is much broader and includes any combination of substance uptake and metabolism, substance metabolism and efflux, or any other interaction in intermediary metabolism or cell signaling.

## Results

Below, we recapitulate the principles of our approach and explain some critical items in the generation of the stable double transfection. The system was then tested and validated by three gene pairs, a pair of two membrane bound fluorescently labeled transport proteins, a pair of two cytosolic fluorescent proteins used particularly to prove the stability of stable expression, and as a pharmacologically relevant application a pair of a membrane transporter (OCT1) and a drug-metabolizing enzyme (CYP2C19).

### Strategy for the Double-Flp-In and the generation of the required vectors

To generate the Double Flp-In system we took advantage from the fact that by the integration of a single plasmid in the classical Flp-In system, the FRT site is not destroyed, but is even duplicated. This enables the integration of at least one additional expression vector in one of the FRT sites.

Therefore, we engineered a second expression vector (pcDNA5/FRT_puro_-fs) carrying a puromycin resistance gene. This additional expression vector was created based on the backbone of the existing pcDNA5/FRT vector by replacing the hygromycin with a puromycin resistance gene (Fig. S1). Additionally, a SV40 promoter region, as present in the host cell genome, was included in the vector to facilitate the expression of the hygromycin resistance gene after chromosomal integration of both vectors. To ensure that this promoter will not drive expression of the puromycin resistance gene in the non-integrated transient state, we generated a reading-frame shift by introducing a single base between the start codon ATG and the puromycin resistance coding sequence. In addition, a one-base deletion was introduced into the original pcDNA5/FRT vector, resulting in the pcDNA5/FRT_hygro_-fs plasmid (Fig. 2). This frame shift was necessary to restore the distance between the ATG from the pcDNA5/FRT_puro_-fs and the hygromycin resistance coding sequence from the pcDNA5/FRT_hygro_-fs. This enables the expression of the hygromycin resistance of the downstream plasmid by the SV40 promoter introduced by the upstream plasmid.

### Transfection protocol optimization

Since the main difference between the transfection protocol for one vector as provided by the manufacturer and our approach is the second vector and the requirement of a second antibiotic, we had to titrate the antibiotics concentrations required to eliminate non-resistant (i.e. not correctly transfected) HEK293 cells by a simple checkerboard approach^20^. The result was a slightly reduced concentration of hygromycin compared to the one typically used for single transfection of HEK293 cells in our laboratory for initial selection of cell clones (200 µg/mL instead of 300 µg/mL). During initial cultivation of cell clones, we also used a reduced hygromycin concentration of 50 µg/mL instead of typically 100 µg/mL. The concentration of puromycin was more difficult to obtain, because the HEK293 cells were highly sensitive to puromycin treatment. The final concentration used for the double transfection was 0.25 µg/mL during selection and 0.025 µg/mL during the cultivation period until complete validation of the cell clones.

Regarding the amounts of DNA used for transfection, initial experiments showed that an increased amount of transfected vector DNA, compared to the single transfection protocol, is not required and only leads to unwanted multiple integrations due to the surplus of vector DNA (data not shown). The ratio of 400 ng pcDNA5 vector(s) to 3.6 µg pOG44 (encoding the transient expression of the Flp recombinase) was maintained as stated in the original transfection protocol by the manufacturer. In case of double transfections, the plasmids were mixed and transfected in an equimolar ratio.

In a preliminary experiment, selection of double-transfected cells was performed with only hygromycin and 22 out of 25 cell clones showed proper expression of both genes of interest (eGFP and tdTomato).

### Double transient vs. double stable transfection

**Figure 3:**
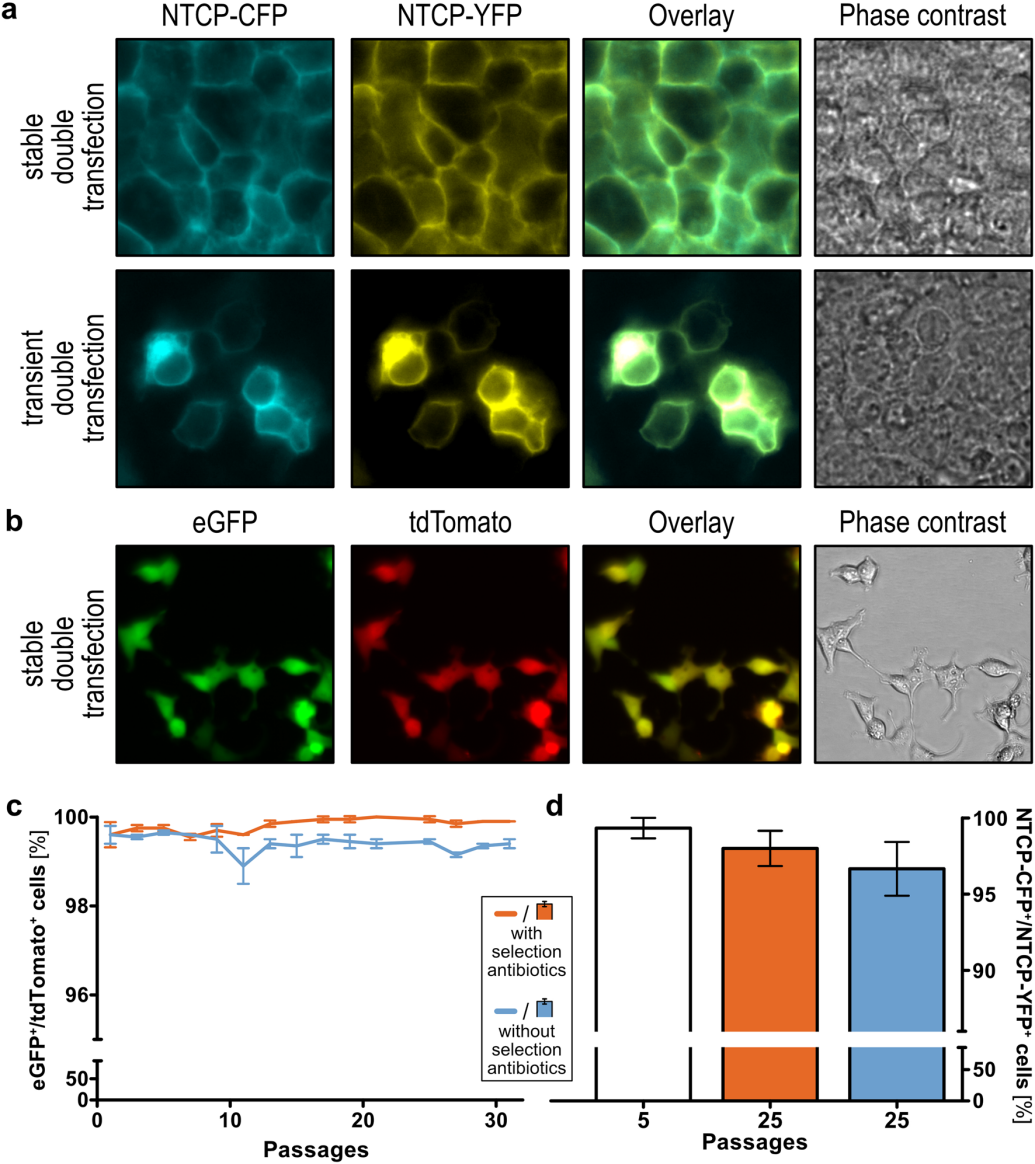
Microscopic and flow cytometric analyses of double-transfected cell lines overexpressing fluorophores. (**a**) Simultaneous co-transfection of the sodium/bile acid cotransporter (NTCP) tagged with cyan or yellow fluorescent protein (CFP, YFP) into HEK293 cells reveals an even expression in virtually all cells. Transient co-transfection of NTCP-CFP and NTCP-YFP illustrates the heterogeneity in strength and distribution of transient transfections, compared to the stably integrated double-transfection. (**b**) HEK293 cells showed consistent overexpression of tdTomato and eGFP during the early selection process by live-cell imaging. (**c**) Flow cytometric analysis of the integration of the genes encoding eGFP and tdTomato showed a high stability over 30 passages (mean ± SEM). (**d**) Stability of genomic integration of simultaneously transfected CFP- and YFP-tagged sodium/bile acid cotransporter (NTCP) was additionally confirmed by microscopic analysis (mean ± SEM). Figures (**c**) and (**d**) were created using GraphPad Prism version 5.01 for Windows, GraphPad Software, La Jolla, California, USA, www.graphpad.com.

For the initial testing of the created plasmids, we double-transfected the cyan or the yellow fluorescent protein (CFP, YFP) tagged sodium/bile acid cotransporter (NTCP) into HEK293 cells and compared the transient to the stable transfection. The fluorescent cells were analyzed by microscopy (Fig. 3a). The stable transfection and clonal selection of cells provided a homogeneous signal with highly comparable signals per individual cell. Moreover, the cells were positive for both transfected fluorescent proteins. On the other side, transiently transfected cells were characterized by a limited efficiency as not all cells showed a fluorescence signal and by a high signal variability within the fluorescent cells, making it difficult to find microscopy settings for an average signal.

### Stability of the genomic integration

The stability of double transfection was determined by flow cytometry and confocal microscopy. Cells validated by flow cytometry had been transfected with pcDNA5/FRT_puro_-fs::tdTomato and pcDNA5/FRT_hygro_-fs::eGFP and double-positivity was compared to empty vector-transfected cells (Fig. 3b). Results showed a stable expression and presence of both fluorescence proteins over 30 passages, regardless of whether the selection antibiotics used for clonal selection was present in the cell culture medium or not. After 25 passages, 99.95% (± 0.03% SEM) of the cells cultivated in medium containing hygromycin and puromycin, and 99.5% (± 0.03% SEM) of the cells cultivated in medium without selection antibiotics were double-positive (Fig. 3c). Similar results were obtained when the stability of the integration was analyzed in the cells double-transfected with CFP- and YFP-tagged NTCP. Here, the signals were analyzed by fluorescence microscopy. After 25 passages, the amount of double-positive cells was not significantly different (unpaired t-test, p > 0.05, GraphPad Prism version 5.01 for Windows, GraphPad Software, La Jolla, California, USA), in cells cultured with and without selection antibiotics (98.0% ± 1.2% SEM and 96.7% ± 1.8% SEM) (Fig. 3d). Altogether, although a very minor decline appeared to exist according to the nominal values (Fig. 3c,d), this decline was not significant.

### Genomic validation

**Figure 4:**
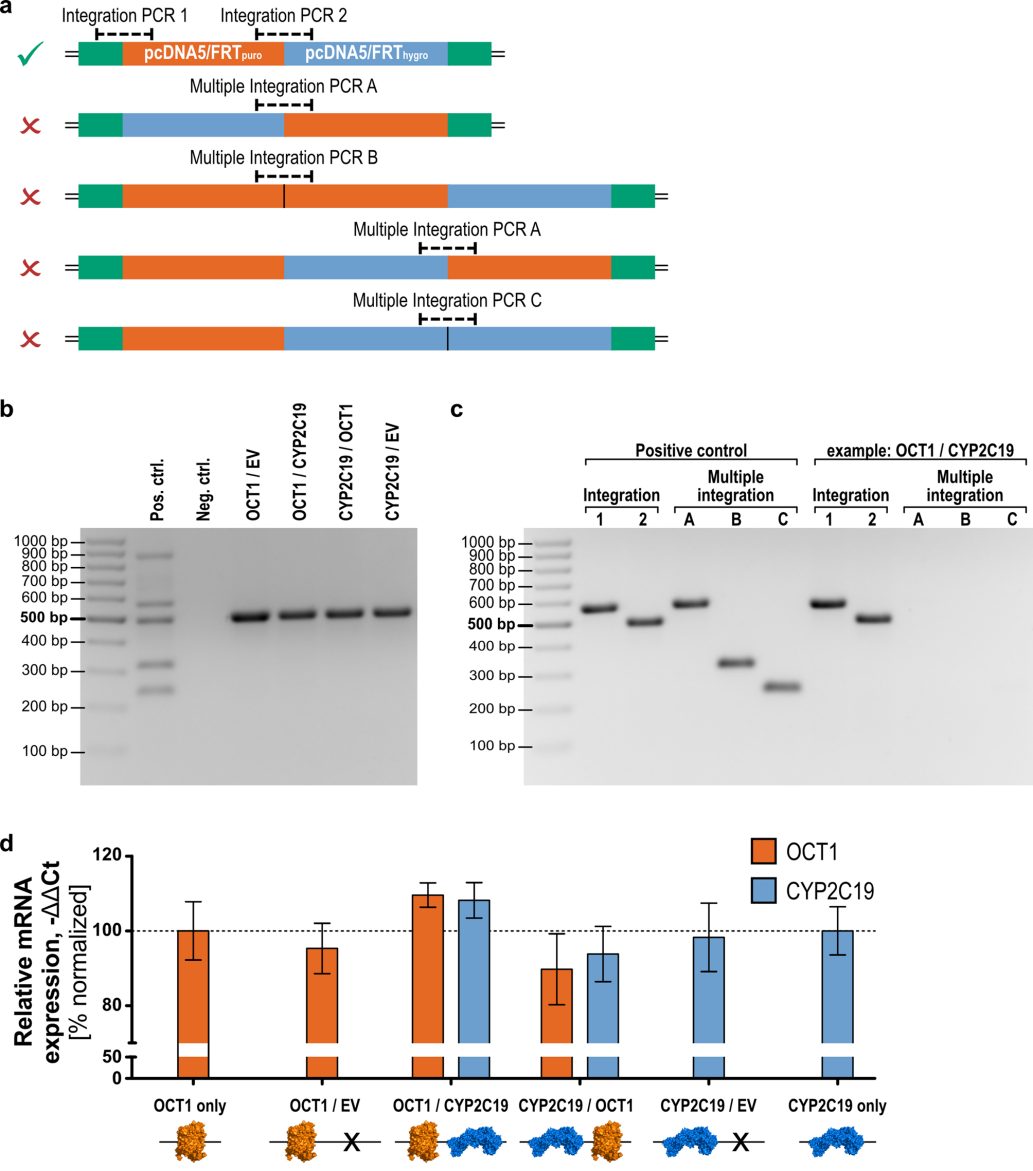
Validation of the double-Flp-In cell clones on genomic and transcriptional level. (**a**) Simultaneous integration of two vectors my lead to at least four unwanted combinations (red ‘x’) besides the intended one (green tick). (**b**) Screening of cell clones by multiplex PCR allowed verification of single integration of each vector (*EV* empty vector; primers as given in Table 1). (**c**) More precise single PCRs were used to validate correct integration for each cell clone, as shown here using one representative example. (**d**) OCT1 and CYP2C19 gene expression analysis of double-Flp-In cell clones compared to single transfected cell lines. Results of n = 3 independent experiments are shown. Gene expression was normalized to the OCT1 only or CYP2C19 only transfected cell lines, respectively. Figure (**d**) was created using GraphPad Prism version 5.01 for Windows, GraphPad Software, La Jolla, California, USA, www.graphpad.com. Protein icons of OCT1 and CYP2C19 were created using The PyMOL Molecular Graphics System, Version 1.3, Schrödinger, LLC, www.pymol.org.

The integration of both vectors in the intended order was confirmed and different cases of multiple integrations were analyzed by PCR. The PCRs were established to detect all possible different arrangements of the two integrated vectors. For this, primers were designed binding at specific elements only present in the FRT locus of the host cell line or in one of the two expression vectors. This led to two reactions confirming the successful integration of both vectors in the intended order and three reactions detecting all setups of multiple integrations (Fig. 4a). This genomic validation assay was planned in a way which allowed to perform four of the reactions simultaneously as part of a multiplex polymerase chain reaction since these four reactions share in total two forward and two reverse primers, and their amplicons differ sufficiently in length. This provides a suitable basis for initial screening of cell lines covering one integration PCR and all three multiple integration PCRs.

Next, we generated OCT1/CYP2C19 overexpressing cell lines in several combinations to perform functional studies later. For each cell line, a number of cell clones were screened by multiplex PCR. The result of this screening is shown in Fig. 4b for all cell clones used for transport experiments in this study. Considering the known limitations of multiplex PCR, we subsequently confirmed the results by performing single PCRs for each reaction and each cell line. Figure 4c shows the exemplary result of this single analysis for one of the analyzed clones which confirmed the findings of the multiplex reaction. Single PCRs for all other double-transfected cell clones used in this study are shown in Fig. S2. Sanger sequencing of the regions around the three FRT sites showed proper arrangement (Fig. S3), as indicated by PCR screening.

### Quantification of gene expression

In a next step, we addressed the question of whether the integration of a second expression vector and the expression of a second gene of interest affects the expression of the first gene of interest and vice versa. For this, we analyzed the gene expression of selected cell clones, which had passed the genomic validation process. In particular, we compared the expression of OCT1 and CYP2C19 of single transfected cell lines to the double transfected ones and moreover analyzed whether the order of integration of both vectors affects the expression of the genes of interest (Fig. 4d). The OCT1 expression levels were highly similar among all analyzed cell lines. In the first place, the number of integrated vectors did not significantly affect the OCT1 expression (One-way ANOVA with post-hoc Tukey test) and secondly, the order of integration as shown by comparison of the OCT1/CYP2C19 (OCT1 in the pcDNA/FRT_puro_ and CYP2C19 in pcDNA/FTR_hygro_) and the CYP2C19/OCT1 (CYP2C19 in the pcDNA/FRT_puro_ and OCT1 in pcDNA/FTR_hygro_) cell lines did also not alter the level of gene expression. The same findings were made for the expression of CYP2C19. Its relative expression in all double-transfected cell lines shows no significant difference in comparison to the CYP2C19 only transfected cell line and the order of integration did not affect its expression as well. Moreover, the basal expression of OCT1 and CYP2C19 genes was negligible (not detected) in empty vector-transfected cell lines (data not shown) and cell lines not overexpressing these genes.

### Functional validation

**Figure 5:**
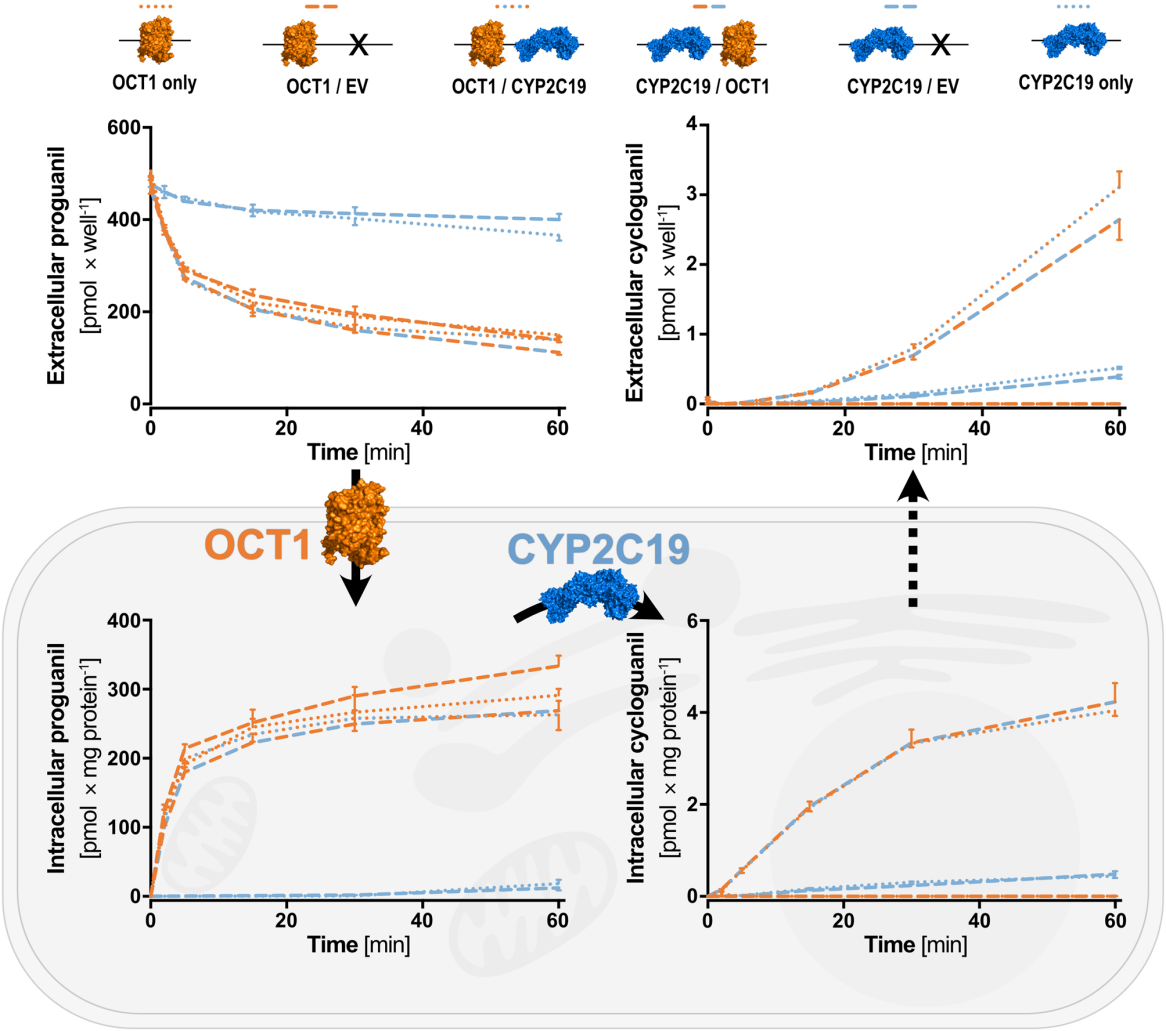
Transport and metabolism of proguanil via two simultaneously transfected genes. Overexpression of OCT1 facilitates the uptake of the anti-malarial prodrug proguanil, while overexpression of CYP2C19 catalyzes the metabolism of proguanil to cycloguanil. Accumulation of cycloguanil was only present in cells overexpressing OCT1 as well as CYP2C19. Results of n = 3 independent experiments are shown. Figure was created using GraphPad Prism version 5.01 for Windows, GraphPad Software, La Jolla, California, USA, www.graphpad.com. Protein icons of OCT1 and CYP2C19 were created using The PyMOL Molecular Graphics System, Version 1.3, Schrödinger, LLC, www.pymol.org.

For the functional validation of this novel overexpression system, we performed cellular uptake and metabolism experiments with the OCT1/CYP2C19 generated cell lines. For this, the uptake via OCT1 and the metabolic activation of proguanil to cycloguanil via CYP2C19 were analyzed (Fig. 5). Both, the transporter and the phase I enzyme were previously known to be involved in the uptake and metabolism of proguanil^21,22^. Uptake of proguanil was remarkably increased in OCT1 overexpressing compared to non-overexpressing cell lines. However, the uptake as indicated by the reduction of extracellular proguanil as well as the accumulation of intracellular proguanil was highly similar in all different OCT1 overexpressing cell lines. This could be regardless of whether the cells were single or double transfected, of the order of integration (OCT1/CYP2C19 or CYP2C19/OCT1) and of whether the second expression vector was empty or carrying CYP2C19. Accumulation of cycloguanil was only observed in cell lines overexpressing CYP2C19. Nevertheless, it was highly increased in double-transfected cell lines co-overexpressing OCT1 as compared to cell lines overexpressing CYP2C19 alone. Additionally, a time-dependent increase of extracellular cycloguanil was also observed as a result of passive diffusion or the presence of endogenous transporters facilitating the export of cycloguanil. Both, the intracellular accumulation as well as the extracellular increase of cycloguanil, were indistinguishable within the two OCT1 and CYP2C19 overexpressing cell lines, but also within the single CYP2C19 and CYP2C19/mock-double transfected cell lines. Pharmacologically, these experiments demonstrate how remarkable a drug metabolic activity in a cell system is enhanced by combined overexpression of the relevant transport protein with the drug-metabolizing enzyme, compared to the enzyme alone.

## Discussion

We established a robust transfection system for simultaneous overexpression of two different genes using several modifications of the Flp-In system^5–8^, and confirmed the long-term stability of gene expression even in the absence of selection antibiotics. We demonstrated that the integration of a second expression vector in the Flp-In locus is not only possible, but also that expression of two genes of interest does not affect each other's expression, and expression levels are highly similar to single-overexpressing cells. Our protocol allows the generation and validation of isogenic cell lines within approximately four weeks. This system provides an ideal tool for studying the interaction of two proteins under well-defined conditions.

As one application from the area of pharmacogenomics pathway analysis, we demonstrated its value for understanding the distinct interactions between transporter mediated drug cell uptake and drug metabolism on the example of the uptake of proguanil and its metabolomic activation to cycloguanil. The obtained results were highly comparable to experiments performed with primary human hepatocytes^22^, indicating our system might be suitable to partially replace drug metabolism analysis in primary human tissue. Moreover, only in the combination with the right transporter, the CYP2C19 overexpressing cells are highly efficient in biotransformation. Such systems may even be further developed for biosynthetic production purposes.

In previous publications, researchers showed the possibilities of multigene expression using the Flp-In system by two succeeding transfections^19^. This approach, however, is not just more laborious, since it requires two consecutive transfections, it also goes along with clear disadvantages, when a single vector backbone is used: the lack of specific selection antibiotics for each gene of interest and the lack of validation on genomic level (and the early exclusion of clones with multiple integrated vectors from the validation process). The new vectors with novel frame shift matches could be conveniently used by other scientists, who used the Flp-In system as one part of their strategy for generating multiple transfectants^23,24^. These projects could profit from the simultaneous transfection of two Flp-In vectors, since the intermediate validation and screening procedure after conventional transfection could be skipped. In contrast to IRES-dependent expression of two genes of interest from a bicistronic vector, there is no effect on the expression level in our system^18^.

Depending on the purpose, the here presented method can easily be modified or refined. The Flp-mediated recombination could be complemented by other recombination enzymes using specific integration sites. Also, modification of the FRT site could modulate specificity of the location of vector integration^25^. This means that several divergent genomic FRT sites could serve as target sites for analogously divergent FRT sites in vector, and recombination could be facilitated by the same enzyme at different sites simultaneously. The frame-dependent expression of the resistance gene only in the case of successful genomic integration is limited to two frames in the case of the FRT site—the third frame leads to a premature stop within the recombination site. Sequence modifications or the use of other recombination enzymes with needs for different recombination sites could solve this issue potentially.

Limitations of the here presented method, such as the treatment with two antibiotics simultaneously, could apply to very sensitive cell lines. However, the use of only hygromycin could be sufficient to select double-transfected cells, because the resistance gene is only expressed after integration downstream to the pcDNA5/FRT_puro_-fs vector. In preliminary experiments, 22 out of 25 cell clones with fluorescent genes of interest showed integration of both vectors after selection with only hygromycin. This might be useful when adapting the here presented protocol to different cell systems. A general problem of stable integration by site-specific recombinases (SSRs) is the integration in pseudosites with high homology to the recombination target site. This off-target activity of SSRs, however, is lower than by other site-specific DNA integration systems^10^.

Double transfections using the method shown here or possible further adoptions to triple or quadruple transfections may serve a scientifically very important bridging between the study of single genes and complex systems biology studies of a large number of genes. Overall we believe that this technique will be helpful in the near future to not only investigate the associations among pharmacogenes, but will be useful when studying gene interactions, and could be extended to three or more genes of interest.

## Methods

### Modification of the original vector pcDNA5/FRT

To match the introduced frame shift between the promoter and the FRT site, the original Flp-In vector pcDNA5/FRT (Thermo Fisher Scientific, Darmstadt, Germany) was modified by deletion of one cytosine (C1590del) and termed pcDNA5/FRT_hygro_-fs here. This modification was achieved by site-directed mutagenesis^26^. For this, 1.0 µL (50 ng) of the pcDNA5/FRT vector was mixed with 5 µL Q-Solution (Qiagen, Hilden, Germany), 2.5 µL 10 × KOD buffer (KOD Hot Start DNA Polymerase Kit; Merck, Darmstadt, Germany), 2.5 µL dNTPs (2 mM each), 1 µL MgSO_4_ (25 mM), 0.65 µL forward primer SDM_hygro_fwd (5′-GTATAGGAACTTCCTTGGCAAAAAGCCTGAACTCACC-3′), 0.65 µL reverse primer SDM_hygro_rev (5′-GGTGAGTTCAGGCTTTTTGCCAAGGAAGTTCCTATAC-3′), 0.5 µL HotStart KOD Polymerase and 11.2 µL H_2_O, and PCR was carried out at 95 °C for 3 min, followed by 20 cycles of 95 °C for 30 s, 63 °C for 45 s, and 72 °C for 4 min. The product was digested with DpnI. For this, 25 µL of the PCR product were mixed with 3 µL cut smart buffer (New England Biolabs, Ipswich, USA) and 1.5 µL DpnI (20 units/mL; New England Biolabs) and incubated at 37 °C for 1 h. After this, further 1 µL DpnI was added and the mixture was incubated at 37 °C for one more hour. The product was dialyzed and transformed into E.coli using an *Electroporator Gene Pulser II* (Bio-Rad Laboratories, Hercules, California, USA) for clonal amplification prior to sequence validation.

### Generation of second vector (pcDNA5/FRT_puro_)

The second vector was built to include the same promoter as in the Flp-In host cell line (from vector pcDNA5/lacZeo, Thermo Fisher Scientific) including the FRT site, the puromycin resistance gene, and the backbone of the regular pcDNA5/FRT vector. To distinguish the two vectors by PCR, we also introduced a unique sequence, at which primers could bind for the eventual validation. For cloning, the three different fragments were generated by PCR using hybrid primers to generate overlapping amplicons (Fig. S1). The fragments of the puromycin resistance gene and the SV40 promoter/FRT region were then fused by overlap-extension PCR^27^ and recombined with the pcDNA5 backbone by sequence and ligation independent cloning^28–30^.

To amplify the pcDNA5 backbone, 10 µL Q-solution (Qiagen, Hilden, Germany), 5 µL 10 × KOD buffer (KOD Hot Start DNA Polymerase Kit; Merck, Darmstadt, Germany), 5 µL dNTPs (2 mM each), 3 µL MgSO_4_ (25 mM), 1.5 µL forward primer PuroR_p5_fwd (5′-cacgaccccatgGGCTGGATGATCCTCCAGCG-3′), 1.5 µL reverse primer SV40/FRT_p5_rev (5′-gacacgtacgtacgtGGCGAACGTGGCGAGAAAGG-3′), 1 µL HotStart KOD polymerase, 1.5 µL pcDNA5/FRT DNA (100 ng) and 21.5 µL H_2_O were mixed and PCR was carried out at 95 °C for 2 min, followed by 35 cycles of 95 °C for 30 s, 68.1 °C for 30 s, 72 °C for 5 min and completed at 72 °C for 10 min. The PCR product was DpnI digested to remove template DNA interfering with subsequent cloning steps as described above. The PCR for the amplification of the SV40 promoter region and the FRT site was composed of 10 µL Q-solution, 5 µL 10 × KOD buffer, 5  µL dNTPs (2 mM each), 3  µL MgSO_4_ (25 mM), 1.5 µL forward primer p5_SV40/FRT_fwd (5′-gccacgtacgtacgtGTCAGTTAGGGTGTGGAAAG-3′), 1.5 µL reverse primer PuroR_SV40/FRT_rev (5′-tcggtggccaagGAAGTTCCTATACTTTCTAGAG-3′), 1 µL HotStart KOD polymerase, 1.5 µL pcDNA5/lacZeo DNA (100 ng) and 21.5 µL H_2_O and carried out at 95 °C for 2 min, followed by 35 cycles of 95 °C for 30 s, 64.9 °C for 30 s, 72 °C for 1 min and completed at 72 °C for 10 min. To amplify the puromycin resistance gene, 10 µL Q-solution, 5 µL 10 × KOD buffer, 5 µL dNTPs (2 mM each), 3 µL MgSO_4_ (25 mM), 1.5 µL forward primer SV40/FRT_PuroR_fwd (5′-ttccttggccACCGAGTACAAGCCCACGG-3′) , 1.5 µL reverse primer p5_PuroR_rev (5′-tcatccagccCATGGGGTCGTGCGCTCC-3′) , 1 µL HotStart KOD polymerase, 1.5 µL of a puromycin resistance gene-containing vector (100 ng) and 21.5 µL H_2_O were mixed and PCR was carried out at 95 °C for 2 min, followed by 35 cycles of 95 °C for 30 s, 68.1 °C for 30 s, 72 °C for 2 min and completed at 72 °C for 10 min.

For the recombination of the ‘SV40 promoter-FRT’ region and the puromycin resistance gene, overlap-extension PCR composed of 10 µL Q-Solution, 5 µL 10 × KOD buffer, 5 µL dNTPs (2 mM each), 3 µL MgSO_4_ (25 mM), 5 µL purified PCR product of SV40/FRT PCR (60 ng/µL), 5 µL purified PCR product of puromycin resistance gene (109 ng/µL; equimolar ratio), 1 µL HotStart KOD polymerase and 17 µL H_2_O was performed at 95 °C for 2 min, followed by 35 cycles of 95 °C for 30 s, 50 °C for 45 s, 72 °C for 2 min and completed at 72 °C for 10 min. Subsequently, 2 µL of the PCR product were mixed with 10 µL Q-Solution, 5 µL 10 × KOD buffer, 5 µL dNTPs (2 mM each), 3 µL MgSO_4_ (25 mM), 1.3 µL forward primer p5_SV40/FRT_fwd (5′-gccacgtacgtacgtGTCAGTTAGGGTGTGGAAAG-3′), 1.3 µL reverse primer p5_PuroR_rev (5′-tcatccagccCATGGGGTCGTGCGCTCC-3′), 1 µL HotStart KOD polymerase and 22.4 µL H_2_O and incubated at 95 °C for 2 min, followed by 35 cycles of 95 °C for 30 s, 50 °C for 45 s, 72 °C for 3 min and completed at 72 °C for 10 min.

The final vector was recombined by sequence and ligation independent cloning^28–30^. For this, 6 µL of the purified pcDNA5/FRT backbone (60 ng) were mixed with 1.44 µL purified product of the overlap-extension PCR (1:3 vector to insert molar ratio), 1 µL 10 × BSA (New England Biolabs, Ipswich, USA), 1 µL 10 × NEB Buffer 2.1 (New England Biolabs), 0.56 µL H_2_O and 0.5 µL T4 DNA polymerase (3 units/µL; New England Biolabs), incubated at 50 °C for 40 s and subsequently kept on ice for 10 min. After this, dialysis was performed and the dialyzed product was electroporated into One Shot TOP10 Electrocomp *E. coli*. Obtained bacterial clones were screened via colony PCR and correct assembly of the plasmid was confirmed by sequencing.

The created plasmid pcDNA5/FRT_puro_ was modified by site-directed mutagenesis to correct the FRT site, since the FRT site present in the provided pcDNA5/lacZeo differs in one nucleotide from the one present in the original pcDNA5/FRT vector, and to introduce a frame shift (C1757ins) matching the reading frame of the pcDNA5/FRT_hygro_-fs vector. For this, 0.6 µL (50 ng) of the pcDNA5/FRT_puro_ vector were mixed with 5 µL Q-Solution, 2.5 µL 10 × KOD buffer, 2.5 µL dNTPs (2 mM each), 1 µL MgSO_4_ (25 mM), 0.65 µL forward primer SDM_puro_fwd (5′-CATGG**C**AGAAGTT***C***CTATTCCGAAGTTCC-3′), 0.65 µL reverse primer SDM_puro_rev (5′-AATAG***G***AACTTCT**G**CCATGGTAGCCTCC-3′), 0.5 µL HotStart KOD Polymerase and 11.2 µL H_2_O and PCR was carried out at 95 °C for 3 min, followed by 20 cycles of 95 °C for 30 s, 58.1 °C for 45 s, 72 °C for 3 min and completed at 72 °C for 10 min. The product was DpnI digested as described above and transformed into E.coli prior to sequence validation. The obtained vector was termed pcDNA5/FRT_puro_-fs hereafter.

### Cloning CYP2C19 from human liver RNA

Human liver total RNA (TaKaRa Bio, Kusatsu, Japan) was used to clone CYP2C19. For this, 1 µL RNA (1 µg) was diluted by adding 16.75 µL RNase-free water, mixed with 1 µL gene-specific primers (5′-GAGGAAAGAGAGCTGCAGGG-3′), incubated at 72 °C for 10 min and subsequently cooled down to room temperature. After this, 6 µL 5 × RT buffer (SuperScript II Reverse Transcriptase Kit; Thermo Fisher Scientific, Darmstadt, Germany), 3.5 µL DTT (0.1 M), 1 µL dNTPs (2 mM each), 0.5 µL RNase inhibitor (40 U/µL) and 0.25 µL SuperScript II Reverse Transcriptase (200 U/µL) were added and reverse transcription was carried out at 42 °C for 1 h before temperature was raised to 75 °C for 15 min. Synthesized cDNA was amplified with primers containing restriction sites for eventual cloning with HindIII and EcoRV (see Table 1). The PCR for the amplification was composed of 10 µL Q-solution, 5 µL 10 × KOD buffer, 5 µL dNTPs (2 mM each), 2 µL MgSO_4_ (25 mM), 1.3 µL forward primer CYP2C19_HindIII_fwd (5′-AAGAGGAGaagcttACCATGGATCCTTTTGTGGTCCTTG-3′), 1.3 µL reverse primer CYP2C19_EcoRV_rev (5′-CATCTGTgatatcTCAGACAGGAATGAAGCACAGC-3′), 1 µL HotStart KOD polymerase, 2.0 µL cDNA template and 22.4 µL H_2_O and carried out at 95 °C for 3 min, followed by 35 cycles of 95 °C for 30 s, 61 °C for 30 s, 72 °C for 2:30 min and completed at 72 °C for 10 min. After amplification, the CYP2C19 was subcloned by TOPO cloning using the TOPO XL PCR Cloning Kit (Thermo Fisher Scientific, Darmstadt, Germany) following the instructions of the manufacturer. Sequencing revealed two single nucleotide polymorphisms: c.99C>T (rs17885098; synonymous) and c.991A>G (rs3758581, p.ile331val). Both polymorphisms define the *1B variant of CYP2C19, which is not associated with changes in its in vitro catalytic activity^31^ and additionally the most abundant variant in the global population^32–34^. It was therefore used for further cloning into the expression vectors and for performing transport and metabolism experiments.

**Table 1:** Primers used for PCR.

**Primers used for generation of pcDNA5/FRT**_**puro**_** vector**			
Fragment	Primer	Sequene (5′–3′)	Amplicon size (bp)
pcDNA5 backbone	PuroR_p5_fwd	cacgaccccatgGGCTGGATGATCCTCCAGCG	3,834
	SV40/FRT_p5_rev	gacacgtacgtacgtGGCGAACGTGGCGAGAAAGG	
Puromycin resistance	SV40/FRT_PuroR_fwd	ttccttggccACCGAGTACAAGCCCACGG	669
	p5_PuroR_rev	tcatccagccCATGGGGTCGTGCGCTCC	
SV40 promotor-FRT region	p5_SV40/FRT_fwd	gccacgtacgtacgtGTCAGTTAGGGTGTGGAAAG	395
	PuroR_SV40/FRT_rev	tcggtggccaagGAAGTTCCTATACTTTCTAGAG	

**Primers used for site-directed mutagenesis**			
Vector	Primer	Sequence (5′–3′)	Amplicon size (bp)
pcDNA5/FRT_hygro_	SDM_hygro_fwd	GTATAGGAACTTCCTTGGC-AAAAAGCCTGAACTCACC	N/A
	SDM_hygro_rev	GGTGAGTTCAGGCTTTTT-GCCAAGGAAGTTCCTATAC	
pcDNA5/FRT_puro_	SDM_puro_fwd	CATGG**C**AGAAGTT***C***CTATTCCGAAGTTCC	N/A
	SDM_puro_rev	AATAG***G***AACTTCT**G**CCATGGTAGCCTCC	

**Primers used for cloning of CYP2C19**			
Reaction	Primer	Sequence (5′–3′)	Amplicon size (bp)
Reverse transcription	CYP2C19_GSP_rev	GAGGAAAGAGAGCTGCAGGG	N/A
Introduction of restriction sites	CYP2C19_HindIII_fwd	AAGAGGAGaagcttACCATGGATCCTTTTGTGGTCCTTG	1516
	CYP2C19_EcoRV_rev	CATCTGTgatatcTCAGACAGGAATGAAGCACAGC	

**Primers used for Validation PCRs**			
Reaction	Primer	Sequence (5′–3′)	Amplicon size (bp)
Integration PCR 1	P_SV40_	AGCTGTGGAATGTGTGTCAGTTAGG	559
	P_puro_r_	CGACGCGCGTGAGGAAGAGTTCTTG	
Integration PCR 2	P_uni_f_	CGTTCGCCACGTACGTACGTGTCAG	489
	P_hyr_r_	CTTCGCCCTCCGAGAGCTGCATCAG	
Multiple Integration PCR A	P_uni_f_	CGTTCGCCACGTACGTACGTGTCAG	564
	P_puro_r_	CGACGCGCGTGAGGAAGAGTTCTTG	
Multiple Integration PCR B	P_FRT_f_	AATCGGGGGCTCCCTTTAGGGTTCC	313
	P_puro_r_	CGACGCGCGTGAGGAAGAGTTCTTG	
Multiple Integration PCR C	P_FRT_f_	AATCGGGGGCTCCCTTTAGGGTTCC	238
	P_hyr_r_	CTTCGCCCTCCGAGAGCTGCATCAG	

**Primers used for quantitative RT-PCR**			
Gene	Primer	Sequence (5′-3′)	Amplicon size (bp)
CYP2C19	P_CYP2C19_f_	CCTGATCAAAATGGAGAAGGAAAAG	99
	P_CYP2C19_r_	TCTGTCCCAGCTCCAAGTAAG	
HPRT1	P_HPRT1_f_	TGACACTGGCAAAACAATGCA	94
	P_HPRT1_r_	GGTCCTTTTCACCAGCAAGCT	
OCT1	P_OCT1_f_	TGTCACCGAAAAGCTGAGCC	96
	P_OCT1_r_	TCCGTGAACCACAGGTACATC	

The 5′-hybrid part of primers used for generation of overlapping amplicons for creation of the pcDNA5/FRT_puro_ vector is shown by nucleotides in small capital letters. The unique sequence introduced into the generated vector is underscored. The newly introduced base into the pcDNA5/FRT_puro_ vector for frame shift generation is marked in bold, the nucleotide substitution for the correction of the FRT site is highlighted by an italic, bold letter. The position of the single nucleotide deletion introduced into the pcDNA5/FRT_hygro_ vector is shown by a hyphen. Endonuclease restriction sites are indicated by lower case letters.

### Transfection protocol

Generally, cell lines used in this study were cultivated in DMEM culture medium (Thermo Fisher Scientific, Darmstadt, Germany) which was supplemented with 10% fetal calf serum (FCS; Thermo Fisher Scientific, Darmstadt, Germany) and penicillin/streptomycin (100 units/mL, 100 µg/mL; Thermo Fisher Scientific, Darmstadt, Germany) unless explicitly described differently. Cells were cultured at 37 °C in a humidified atmosphere (5% CO_2_, 95% relative humidity). All experiments were carried in HEK293 T-REx cells (Thermo Fisher Scientific, Darmstadt, Germany).

For transfection of HEK293 T-REx cells, one million cells were plated for each transfection in a 6-well plate 24 h in advance. On the day of transfection, for double transfections, 200 ng of each expression plasmid in midi-prep quality were mixed with 3.6 µg pOG44 encoding the Flp recombinase in 100 µL DMEM. For single transfections, 400 ng of expression plasmid was mixed with 3.6 µg pOG44 encoding the Flp recombinase in 100 µL DMEM. Additionally, 12 µL *FuGene 6* transfection reagent (Promega Corporation, Walldorf, Germany), were added to 100 µL DMEM. After an incubation period of 5 min at room temperature, both solutions were mixed by repetitive pipetting and incubated for another 15 min at room temperature. In the meantime, the cells were washed once with 2 mL DMEM with 10% FCS and 1.8 mL fresh DMEM with 10% FCS were added to the cells. After incubation, the 200 µL DNA-*FuGene 6* was pipetted dropwise onto the cells. After 24 h, the cell culture medium was replaced by DMEM with 10% FCS and penicillin–streptomycin. On the next day (48 h post transfection), cells were transferred to a 100 mm petri dish. After 24 h, the selection was performed by adding hygromycin B (final concentration 200 µg/mL; Thermo Fisher Scientific, Darmstadt, Germany) and puromycin (final concentration 0.25 µg/mL; Thermo Fisher Scientific, Darmstadt, Germany). Five days later, the supernatant was replaced by fresh cell culture medium containing hygromycin and puromycin. Around ten days after selection started, single colonies were picked. For this, cells were washed once with culture medium to remove dead cells and finally the medium was completely removed. Single colonies were resuspended in 2 µL medium and transferred to a 24-well-plate, where 1 mL culture medium with a reduced concentration of the selection antibiotics (hygromycin: 50 µg/mL, puromycin: 0.025 µg/mL) had been placed in advance. After reaching a confluence of 70–80%, cells were transferred to a 6 well plate and later to a T25 culture flask. When cells were passaged the first time, 40% of cells were used to prepare cell pellets for DNA and RNA extraction each, to allow validation of generated cell lines on genomic as well as on transcriptional level. Transfections were usually carried out in duplicates to ensure a sufficient number of clones to analyze.

The same protocol was independently performed in the Institute of Pharmacology and Toxicology at the Justus Liebig University in Giessen to stably transfect two versions of the sodium/bile acid transporter (NTCP) tagged with yellow or cyan fluorescent protein (NTCP-YFP, NTCP-CFP).

### Validation of correct integration by PCR

The stable, genomic integration of both plasmids was validated by PCR. For this, the genomic DNA of 2 × 10^6^ cells was isolated using the QIAGEN DNeasy Blood & Tissue Kit (Qiagen, Hilden, Germany) in a QIACube robot (Qiagen) following the manufacturer’s protocol. The isolated DNA was analyzed by multiplex PCR using the QIAGen Multiplex PCR Kit (Qiagen). This reaction covered the *Integration PCR 2* as well as the *Multiple Integration PCRs A-C* (for detailed primer information see Table 1). Multiplex PCR was composed of 5 µL 2 × QIAGEN Multiplex PCR Master mix, 2 µL Q-Solution, 1 µL 10 × primer mix (2 µM each), 1 µL genomic DNA (100 ng) and 1 µL H_2_O. PCR was carried out at 95 °C for 15 min, followed by 35 cycles of 95 °C for 30 s, 62.7 °C for 90 s, 72 °C for 90 s and completed at 72 °C for 10 min. As a positive control, the genomic DNA of a cell clone validated for all types of multiple integrations was used. Cell clones passing the multiplex PCR screening were validated again by single PCRs (*Integration PCRs 1* and *2, Multiple Integration PCRs A-C*) composed of 5 µL 2 × QIAGEN Multiplex PCR Master mix, 2 µL Q-Solution, 0.25 µL forward primer (10 µM), 0.25 µL reverse primer (10 µM), 1 µL genomic DNA (100 ng) and 1.5 µL ddH_2_O using the same thermocycler conditions.

Sanger sequencing was performed on the products including the FRT sites. For the third FRT site, the primers P_FRT_f_ and P_LacZ_ (5′-CCTTCCTGTAGCCAGCTTTCATCAA-3′) under the same conditions as mentioned for single PCRs above. The resulting amplicons were separated on a 0.8% agarose gel, cut out and extracted using the QIAquick Gel Extraction Kit (Qiagen, Hilden, Germany). For sanger sequencing, 100 ng DNA were premixed with 30 pmol of one of the primers used for amplification and sent to external sequencing by Microsynth Seqlab, Göttingen.

### Tracking of eGFP/tdTomato double-transfected cells by flow cytometry

The stability of stable transfection was tracked by analyzing fluorescence signals of eGFP/tdTomato double-transfected cell lines over 30 passages. Two independently generated cell lines were cultured in parallel in DMEM cell culture medium with or without the culturing concentrations of hygromycin (50 µg/mL) and puromycin (0.025 µg/mL). Every second passage, cells were analyzed using a LSR II (BD Bioscience, Heidelberg, Germany) flow cytometer and the software BD FACSDiva (Version 6.1.3, BD Bioscience). Fluorescence intensities of green channel (laser excitation wavelength 488 nm) and red channel (laser excitation wavelength 561 nm) were plotted to determine double-positive cells. Thresholds for classifying cells as positive were set by comparing the fluorophore expressing cells to a mock-transfected control cell line.

### Expression analyses

The RNeasy Plus Mini Kit (Qiagen, Hilden, Germany) was used according to the manufacturer’s instructions for total RNA isolation. Briefly, 1 to 2 × 10^6^ cells were harvested by centrifugation at 500×*g* for 5 min at RT. The pellet was dissolved in 350 µL of RLT buffer supplemented with 1% β-mercaptoethanol (v/v). The automatic isolation was performed using a QIAcube (Qiagen, Hilden, Germany), in which the genomic DNA eliminator spin column removed the genomic DNA and total RNA was eluted in 50 µL of RNAse free ddH_2_O.

The cDNA synthesis from isolated RNA was performed using the SuperScript II Reverse Transcriptase Kit (Thermo Fisher Scientific, Darmstadt, Germany). Three µg RNA was diluted in 17.75 µL of RNAse free ddH_2_O. Primer annealing was initiated with the addition of 1 µL anchored-dT primer (10 µM) and incubation at 70 °C for 10 min. To initiate cDNA synthesis, 11.25 µL of a reverse transcription reaction mix [6 µl 5 × Superscript RT buffer, 3.5 µL DTT (0.1 M), 1 µL dNTPs (10 mM), 0.5 µL RNase Inhibitor P/N (40 U/µL), 0.25 µL SuperScript II Reverse Transcriptase (200 U/µL)] were added and incubated at 42 °C for one hour. Afterward, enzyme denaturation was done by increasing the temperature to 75 °C for 15 min. To this 30 µL synthesized cDNA, 70 µL of RNAse free ddH_2_O were added and concentration was further adjusted to 3 ng/µL cDNA by 1:10 dilution.

HOT FIREPol EvaGreen qPCR Mix Plus (ROX) kit (Solis BioDyne, Tartu, Estonia) was used to perform the real-time qPCR. Briefly, the reaction mixture constituted 2 µL 5 × EvaGreen qPCR Mix, 5.6 µL ddH_2_O, 0.4 µL primer mix (10 µM each; HPRT1: forward (5′-TGACACTGGCAAAACAATGCA-3′), reverse (5′-GGTCCTTTTCACCAGCAAGCT-3′); OCT1: forward (5′-TGTCACCGAAAAGCTGAGCC-3′), reverse (5′-TCCGTGAACCACAGGTACATC-3′), CYP2C19: forward (5′-CCTGATCAAAATGGAGAAGGAAAAG-3′), reverse (5′-TCTGTCCCAGCTCCAAGTAAG-3′)), 2 µL cDNA (6 ng total). Standard curve analysis for each primer pair was performed to check the primer efficiency and amplification of a single specific amplicon. To do so, five concentrations of a cDNA pool in a 1:5 dilution series were distributed in a 384 well-plate and amplification was performed in TaqMan 7900HT (Applied Biosystems, Darmstadt, Germany) machine. SDS 1.2 software (Applied Biosystems, Darmstadt, Germany) was used to identify the Cycle threshold (Ct) value. The Primer efficiency was well within the accepted range, namely 107% (HPRT1), 101% (OCT1), and 99% (CYP2C19)^35^. Subsequently, expression levels of OCT1 and CYP2C19 genes, along with the housekeeping gene HPRT1, were measured in technical and biological triplicate manner. The ΔΔCt method was used for expression analysis^36^. Relative expression against single transfected OCT1 and CYP2C19 cell lines were calculated based on this equation:

$${\text{Relative}}\;{\text{ expression }} = { 2}^{{ - \left[ {({\text{Ct}}\;{\text{ experimental }}{-}{\text{ Ct }}\;{\text{housekeeping }}\;{\text{experimental}}) \, {-} \, ({\text{Ct }}\;\;{\text{control }}{-}{\text{ Ct }}\;{\text{housekeeping }}\;{\text{control}})} \right]}} = { 2}^{{ - [\Delta \Delta {\text{Ct}}]}}$$

### Transport experiments

Functional validation of the stably integrated genes was performed via transport and metabolism of proguanil. Two days ahead of the transport experiment, 600,000 cells were plated in poly-d-lysine pre-coated 12-well-plates. On the day of experiment, cells were washed once with 2 mL 37 °C pre-warmed Hanks buffered saline solution (Thermo Fisher Scientific, Darmstadt, Germany) containing 10 mM HEPES (Sigma-Aldrich, Taufkirchen, Germany; from here on named HBSS+) followed by incubation with HBSS+ containing 1 μM Proguanil (Sigma-Aldrich, Taufkirchen, Germany). After 2, 5, 15, 30 and 60 min, the incubation was stopped by collecting the supernatant and cells were immediately washed twice with 1 mL ice-cold HBSS+ and cells were lysed by adding 500 μL lysis buffer [acetonitrile:water 4:1 (v/v)] containing 10 ng/μL proguanil-d6 (Toronto Research Chemicals, North York, Canada) and 10 ng/μL desvenlafaxine (Sigma-Aldrich, Taufkirchen, Germany) as an internal standard for mass spectrometry analysis. For sample preparation, cell lysates were centrifuged in a desktop centrifuge at maximum speed for 15 min, 400 μL were transferred into a collection plate, evaporated at 40 °C under nitrogen flow and the dry residues were dissolved in 250 μL 0.1% formic acid. For the processing of cell supernatant, samples were centrifuged at 400×*g* for 5 min. After this, 400 μL was transferred to a new reaction tube and 800 μL of precipitation reagent [acetonitrile:methanol 10:1 (v/v) containing the same internal standards as described above] were added and the mixture was incubated for 15 min on a rotation shaker. Precipitated protein was pelleted by centrifugation at full-speed in a desktop centrifuge for 15 min, 800 μL of the supernatant was used for evaporation and dry residues were dissolved in 250 μL 0.1% formic acid for analysis as well.

### LC–MS/MS determination of proguanil transport and metabolism

Concentrations of proguanil and cycloguanil were quantified by high-performance liquid chromatography coupled to mass spectrometry. For sample separation, we used a Shimadzu Nexera 2 UHPLC system containing an auto-sampler SIL-30AC, a communications bus module CBM-20A, a liquid chromatograph LC-30AD, a column oven CTO-20AC and a Brownlee SPP RP-Amide column (4.6 × 100 mm inner dimension with 2.7 μm particle size) with a C18 pre-column. For chromatography, an aqueous mobile phase containing 20% organic additive (acetonitrile:methanol 6:1 (v/v)) was used with a flow gradient starting with 0.3 mL/min for the first 4.5 min, increased to 0.7 mL/min at 4.6 min and back to 0.3 mL/min from 9.0 to 9.1 min, which was left for another two minutes to reconstitute the original conditions for the next measurement. The HPLC system was coupled with an API 4000 tandem mass spectrometer (AB SCIEX, Darmstadt, Germany), which enabled the detection of substrates via specific LC retention times and mass transitions in MRM mode with parameters given in Table S1. The quantification was performed by integration of the peak areas using the Analyst software (Version 1.6.2, AB SCIEX, Darmstadt, Germany). Concentrations of proguanil and cycloguanil (Santa Cruz Biotechnology, Heidelberg, Germany) were determined by simultaneous measurement of a standard curve with known concentrations. To calculate the net uptake of proguanil, the measurement of an empty vector-transfected cell line was subtracted from the other cell lines to take passive diffusion and endogenous transporters into account.

### Total protein quantification

Results of cellular transport experiments were normalized to the total amount of protein per well to compensate differences in cell density. For this, the total protein of one well per cell line in each transport experiment was quantified using a BCA assay. Cells were lysed by incubation with 500 μL RIPA buffer for 10 min. Five microliter of each sample were incubated after adding 200 μL bicinchoninic acid with 0.008% copper sulfate at 37 °C for 30 min. Subsequently, the absorbance at a wavelength of 570 nm was measured using a *Tecan Ultra microplate reader* (Tecan Group, Männedorf, Switzerland). The protein concentration was quantified by comparison to a standard curve using bovine serum albumin.

### Tracking of double-transfected cells by microscopy

HEK293-Flp-In cells stably transfected by our Double-Flp-In Method with NTCP-CFP and NTCP-YFP, a classical Foerster Resonance Energy Transfer (FRET) pair, were seeded onto IBIDI chamber-slides to reach confluency at the day of microscopy. For comparison HEK293-MSR cells were seeded like above and were transiently transfected with Lipofectamine 2000 (Thermo Fisher Scientific, Darmstadt, Germany) with an equimolar number of premixed plasmids of pcDNA5 vectors coding for NTCP-CFP or NTCP-YFP both under the control of the identical CMV-promoter, which is also applied by the Double-Flp-In Method. After 48 h of standard incubation, slides were washed twice with PBS and transferred to microscopy at room temperature covered in PBS. Images were taken with a Leica DMI6000 B inverted fluorescent microscope at 40× objective magnification. For qualitative analysis and comparison of expression levels and patterns of the fluorescent proteins CFP and YFP channels were adjusted to yield similar signal intensities. Phase contrast channel was applied to demonstrate the confluency of the cell layer and transfection rates. Staining of the cell nuclei and the fixation of the cells was deliberately avoided to not interfere with CFP and YFP signals.

## Supplementary information

Supplementary Information.
