# Supplementary material for: A double-Flp-in method for stable overexpression of two genes
Source: Sci Rep. 2020 Aug 20;10:14018. doi: 10.1038/s41598-020-71051-5 (PMC7441062; doi:10.1038/s41598-020-71051-5)
Supplement: Supplementary file 1 — Supplementary Information. [file 41598_2020_71051_MOESM1_ESM.pdf]

Supplementary Information for:

A Double-Flp-In Method for Stable Overexpression of Two Genes

Ole Jensen[1#*], Salim Ansari[1#], Lukas Gebauer[1#], Simon F. Müller[2], Kira A. A. T. Lowjaga[2], Joachim Geyer[2], Mladen V. Tzvetkov[1,3], and Jürgen Brockmöller[1]

[#] These authors contributed equally to the work.

[1] Institute of Clinical Pharmacology, University Medical Center Göttingen, D-37075 Göttingen, Germany

[2] Institute of Pharmacology and Toxicology, Faculty of Veterinary Medicine, Justus Liebig University Giessen, D-35392 Giessen, Germany

[3] Institute of Pharmacology, Center of Drug Absorption and Transport (C_DAT), University Medical Center Greifswald, D-17489 Greifswald, Germany

Address for correspondence:

Ole Jensen
Institute of Clinical Pharmacology
University Medical Center Göttingen, Georg-August University
Robert-Koch-Str. 40
37075 Göttingen, Germany
E-Mail: ole.jensen@med.uni-goettingen.de
Telephone: +49 551 39 65776
Fax: +49 551 39 12767

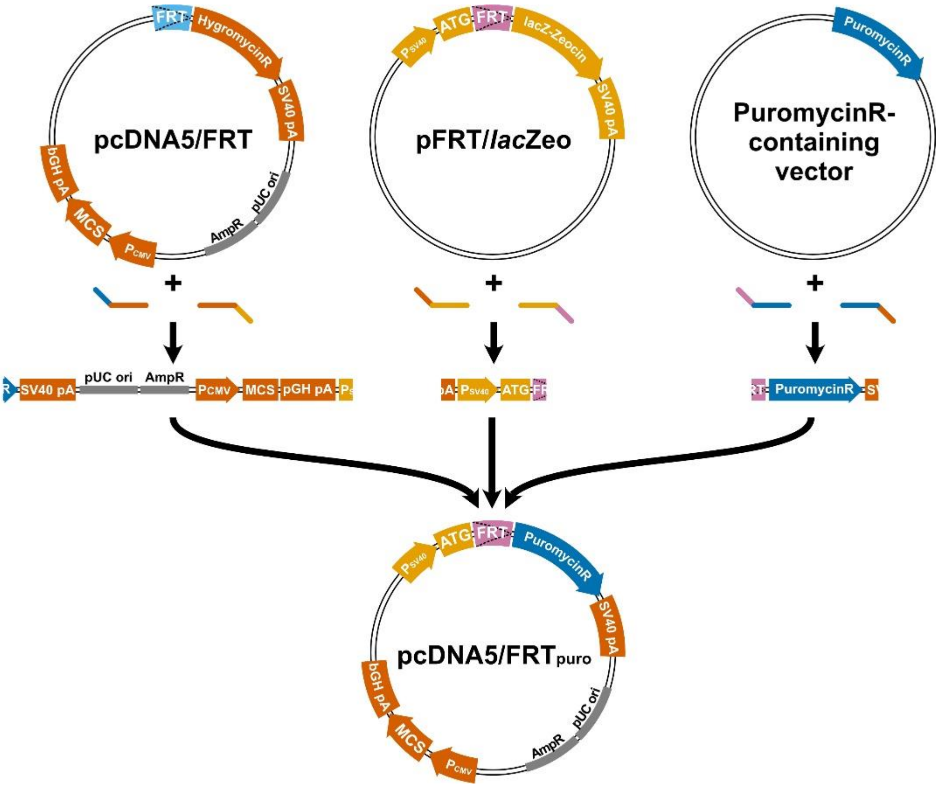

Figure S1: Illustration of vector creation. The newly generated vector pcDNA5/FRT$_{puro}$ was created by PCR amplification with primers including overhangs from two of the vectors that belong to the Flp-In™ system, pcDNA5/FRT and pFRT/lacZeo. The puromycin resistance cassette was cloned from a third puromycin resistance-containing vector by PCR amplification and sequence and ligation-independent cloning (SLIC).

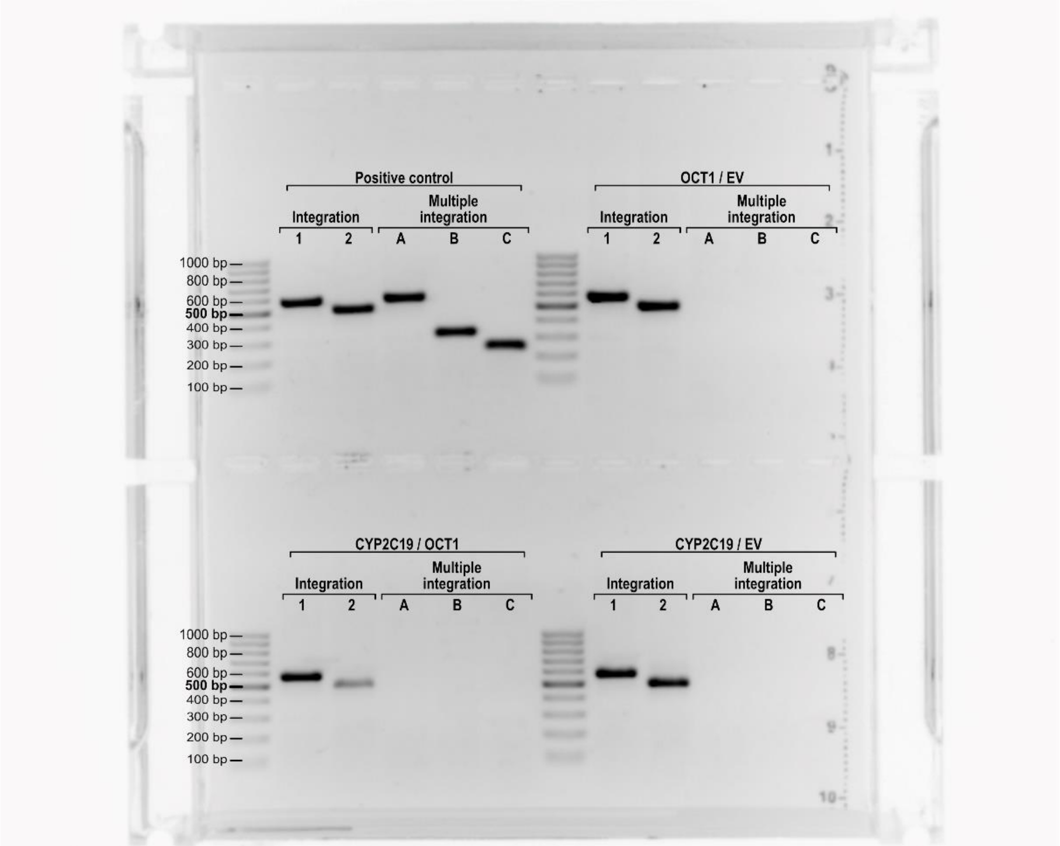

Figure S2: Validation of used cell clones by PCR. In addition to the initial screening by multiplex PCR (see Figure 4), the finally employed cell clones (OCT1/EV, CYP2C19/OCT1, CYP2C19/EV) were validated by single PCRs.

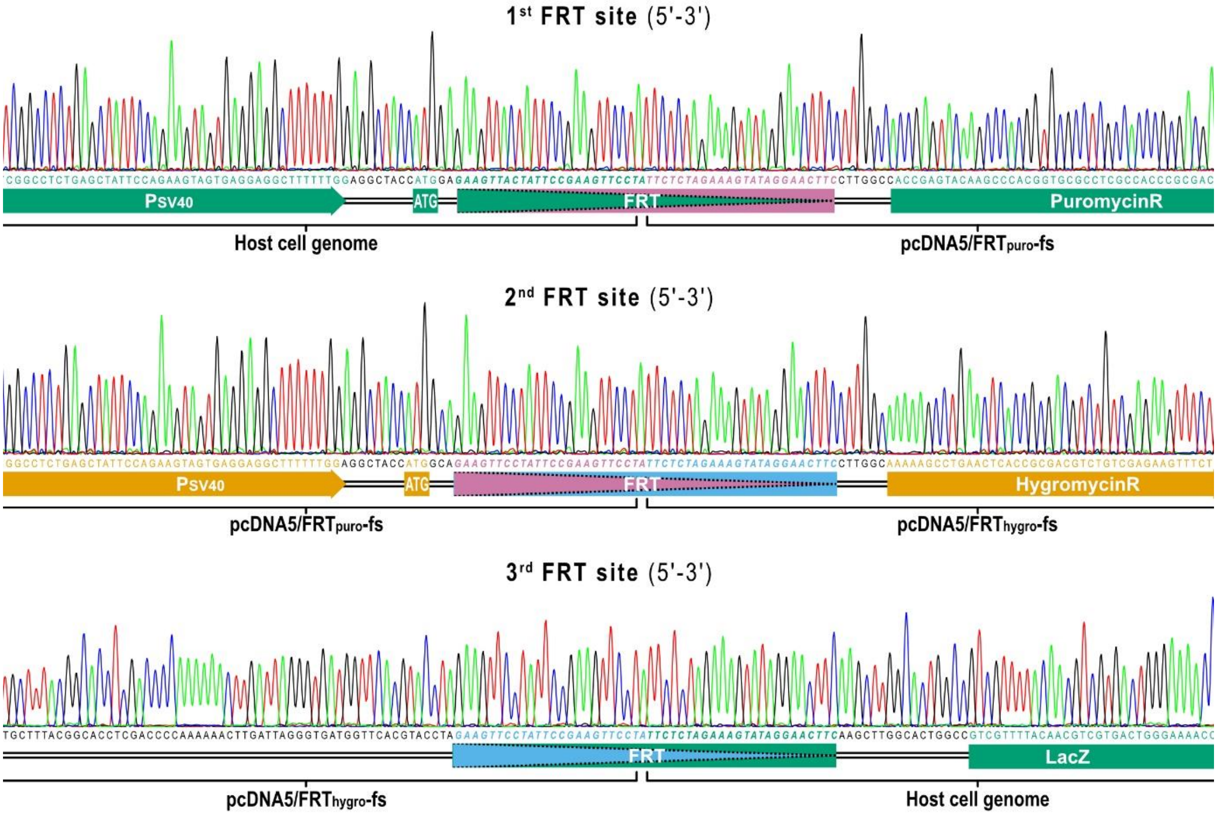

Figure S3: Sequence validation of the three FRT sites including flanking regions. Sanger sequencing was performed on the cell clone OCT1/CYP2C19 to validate correct integration on nucleotide level. Sequences of the FRT sites are highlighted in ***bold italic***, while other functional elements are indicated in their respective color, as shown in Figure 2.

Table S1: Mass spectrometry detection parameters

Substance	Retention time (min)	Mass Q1 (Da)	Mass Q3 (Da)	DP (V)	CE (V)	CXP (V)
Cycloguanil	4.2	252.2	195.1 (153.0)	75	25 (41)	10 (10)
O-Desmethyl-venlafaxine	4.0	264.3	58.1 (107.2)	75	47 (50)	10 (10)
Proguanil	8.7	254.2	170.2 (153.1)	75	24 (40)	10 (10)
Proguanil-d6	8.6	260.3	170.2 (153.1)	75	25 (41)	10 (10)

DP - declustering potential; CE - collision energy; CXP - collision cell exit potential

Figure 4b

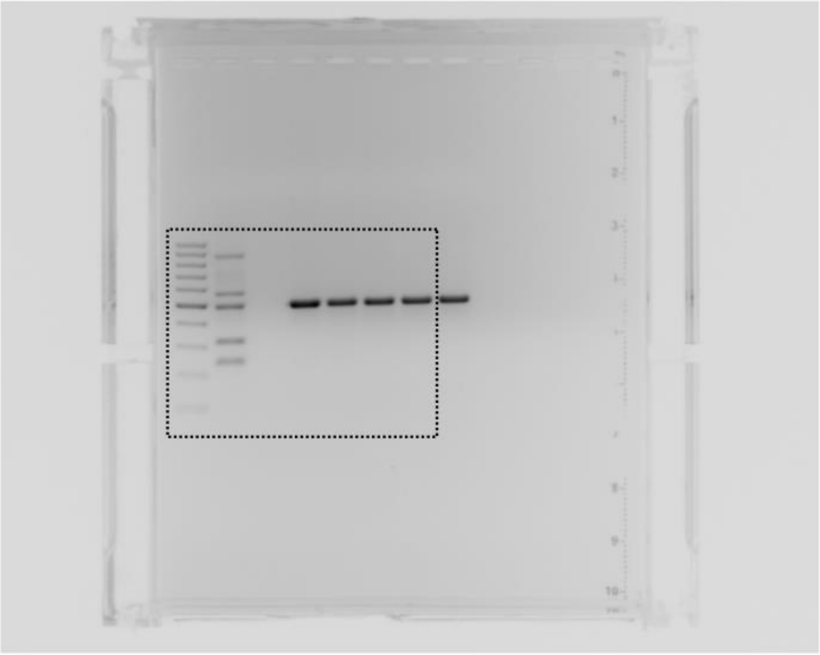

Original gel: Fig. 4b (additional band shows amplification of EV / EV control by multiplex PCR)

Figure 4c

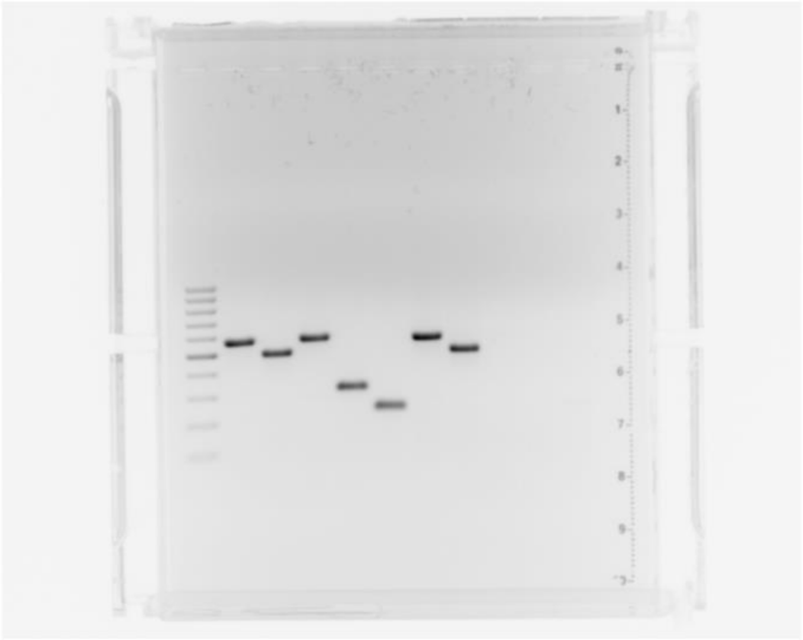

Original gel: Fig. 4c
